# Proprioception as a sensory root for body and motor awareness

**DOI:** 10.1093/braincomms/fcaf379

**Published:** 2025-10-01

**Authors:** Gerardo Salvato, Giulia Casile, Silvia Amaryllis Claudia Squarza, Mariangela Piano, Maria Sessa, Gabriella Bottini

**Affiliations:** Department of Brain and Behavioral Sciences, University of Pavia, Pavia 27100, Italy; Cognitive Neuropsychology Centre, ASST ‘Grande Ospedale Metropolitano Niguarda’, Milano 20162, Italy; NeuroMi, Milan Center for Neuroscience, Milano 20126, Italy; Department of Brain and Behavioral Sciences, University of Pavia, Pavia 27100, Italy; Neuroradiology Unit, ASST ‘Grande Ospedale Metropolitano Niguarda’, Milano 20162, Italy; Neuroradiology Unit, ASST ‘Grande Ospedale Metropolitano Niguarda’, Milano 20162, Italy; Neurology and Stroke Unit, ASST ‘Grande Ospedale Metropolitano Niguarda’, Milano 20162, Italy; Department of Brain and Behavioral Sciences, University of Pavia, Pavia 27100, Italy; Cognitive Neuropsychology Centre, ASST ‘Grande Ospedale Metropolitano Niguarda’, Milano 20162, Italy; NeuroMi, Milan Center for Neuroscience, Milano 20126, Italy

**Keywords:** body ownership, DSO, right-brain damage, motor awareness, anosognosia for hemiplegia

## Abstract

The primary function of proprioception is to stabilize and protect the body. However, its role may extend beyond this fundamental function. Proprioceptive signals have been hypothesized to contribute to the emergence of body awareness (i.e. sense of ownership) and motor awareness (i.e. sense of agency). However, this hypothesis has never been explicitly empirically tested in neuropsychological studies involving a large sample of patients. We investigated this assumption in the context of pathological disturbances of body and motor awareness following right-hemisphere stroke. In an observational, cross-sectional study, we tested 46 consecutive patients assessing proprioceptive abilities, disturbed sensation of ownership (DSO), and anosognosia for hemiplegia (AHP). Our findings reveal that reduced proprioceptive abilities predict both DSO and AHP for the contralesional plegic upper limb. Advanced lesion mapping indicates that the proprioceptive deficit predicting the presence of DSO and AHP is associated with lesions to the right parietal cortex and underlying white matter tracts. The lesional pattern was further detailed by bilateral disconnections between the temporal and parietal areas associated with decreased proprioceptive ability. We discuss the theoretical implications of these results in relation to the ongoing debate on the role of proprioception in bodily self-awareness.

## Teaser

Salvato *et al.* report that proprioceptive deficits predict both disturbed sensation of ownership and anosognosia for hemiplegia following right-hemisphere stroke. Lesion mapping reveals damage to parietal regions and interhemispheric disconnections, supporting a key role of proprioception in both body and motor awareness.

## Graphical Abstract

**Graphical Abstract:**
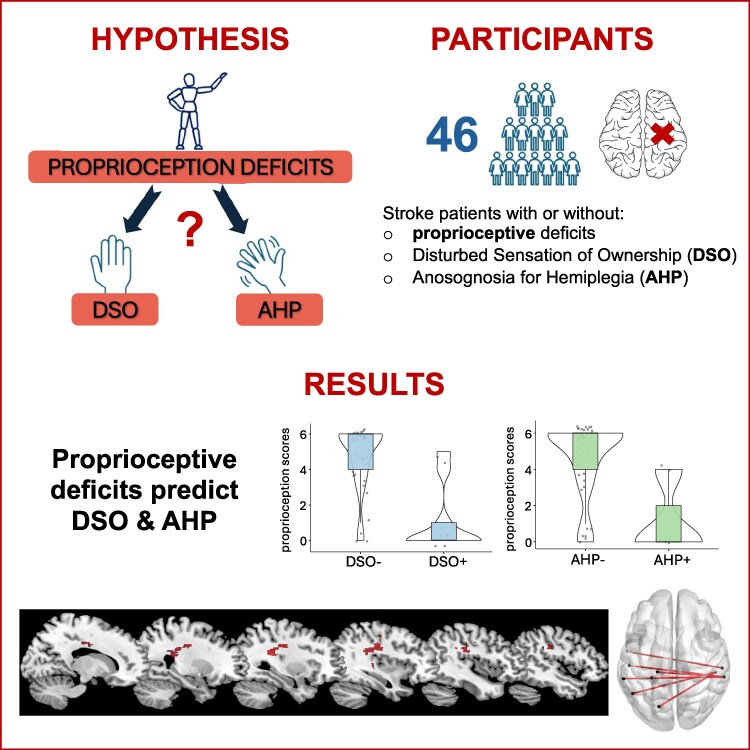

## Introduction

Proprioception is the ability to perceive the position, movement, and balance of the body in space. Often called the ‘sixth sense,’ it is a crucial constituent of the somatosensory system. This ability depends on feedback from specialized mechanoreceptors, known as proprioceptors, which are found in muscles, tendons and joints.^1,2^ Although the neural basis of proprioception remains poorly understood, it is likely supported by a widespread cerebral network. A proposed pathway for ascending proprioceptive feedback is the dorsal column-medial lemniscus system, which is believed to convey consciously accessible proprioceptive information. Proprioceptive afferents may directly or indirectly target the dorsal column nuclei in the brainstem, which then project to thalamic circuits that innervate the cerebral cortex.^2^ These higher-order, broadly distributed processes send information to the somatosensory system, producing finely detailed proprioceptive representations and contributing to the formation of bodily self-awareness. Bodily self-awareness has been defined as the feeling that conscious experiences are bound to the self and are experiences of a unitary entity.^3–7^ It is a multidimensional construct, encompassing various components such as the sense of body ownership, the perception of internal bodily signals, the awareness of the body in space and the sense of being the actor of our own actions.^3–5^

Although the most elementary function of proprioceptive feedback is to stabilize and protect the body, it has been hypothesized that proprioception may also represent the common ground for body awareness (i.e. the sense of ownership) and motor awareness (i.e. the sense of agency), two crucial components of bodily self-awareness. In particular, the sense of ownership concerns the feeling of one's own body as belonging to oneself and the feeling that a given body part belongs to one's body^8,9^ while the sense of agency refers to the feeling of controlling one’s movements, through them, the events in the outside world.^10,11^ It is increasingly recognized that the sense of ownership and the sense of agency refer to specific cognitive constructs underlying the bodily self-awareness experience, and several studies have investigated their relationship at both behavioural and neural levels. Although the most recent evidence has emphasized the interrelationship between body and motor awareness,^12^ the common ground is still opaque. Some interesting suggestions came from Synofzik *et al.*,^13^ which identified proprioceptive abilities specifically as the link between ownership and agency.

Only a few correlational studies have explored the role of proprioception in body and motor awareness, albeit indirectly. Pyasik *et al.*^14^ assessed the relationship between implicit and explicit measures of the sense of ownership and agency through the rubber hand illusion and Libet’s clock paradigms, respectively. They found that the proprioceptive drift, although operationalized as an implicit measure of body ownership, was positively correlated with an implicit measure of agency, namely, auditory attenuation. Shibuya *et al.*^15^ did not find consistency with these results, suggesting that the sense of ownership and agency influence proprioceptive drift based on task demand. Nevertheless, in another study,^16^ while testing the relationship between the sense of ownership and sense of agency by incorporating the intentional binding task into the rubber hand illusion paradigm, the authors did not find any correlation between explicit experiences of ownership and agency with the proprioceptive drift.

While the available behavioural evidence in healthy populations, framed within a different theoretical perspective, remains indirect, correlational and often conflicting, the complexity increases further when exploring the neural correlates of proprioceptive signals that contribute to body and motor awareness. Most of our current understanding stems from studies that have examined proprioception separately in the framework of body or motor awareness. For instance, two recent meta-analyses have found anatomical overlap between proprioception and body ownership in the parietal cortex. Grivaz *et al.*^17^ found a cluster in the intraparietal sulcus related to rubber hand illusion paradigms. Similarly, Salvato *et al.*^18^ found that the right parietal cortex was an area of convergent activation across rubber hand illusion studies, a region that has been associated with the real hand position remapping onto a prosthetic hand.^19^ The parietal cortex is also an important region potentially subserving the relationship between proprioception and motor awareness. For instance, the posterior parietal cortex has been considered a central hub in integrating proprioceptive feedback with motor actions, which is crucial for maintaining a coherent sense of agency.^20^ Additionally, the inferior parietal lobe has been considered a crucial area for constructing a unified body schema by integrating proprioceptive information with motor intentions.^21^

In sum, in healthy subjects, the causal role of proprioception in body and motor awareness is mainly grounded on fragmented findings. The indirect, partial and only correlational evidence on the topic and the absence of a tailored behavioural and neuroimaging study contribute to the limited understanding. In this framework, studying patients with focal brain damage can contribute to a causal and explicative model of the role of proprioception in the interaction between body and motor awareness. Indeed, through the neuropsychological lesion-behaviour approach applied to the model of proprioception and body and motor awareness disorders, we can provide behavioural and neural causal evidence on this complex relationship. Proprioception, body and motor awareness may deteriorate in many neurological conditions. In the case of right-hemisphere stroke, patients may present difficulty in localizing the left contralesional hand in space. Proprioceptive impairment occurs in up to 64% of individuals after stroke.^22^ Right-brain stroke may also alter body awareness, provoking the so-called ‘disturbed sensation of limb ownership’ (DSO).^23^ DSO is a label mainly used in research settings that clusters symptoms, including the feeling of non-belonging or non-recognition of the contralesional plegic limb (asomatognosia^24^) and/or delusional ideas of disownership (somatoparaphrenia).^25,26^ Lastly, patients with right brain damage and left-hand hemiplegia may present with unawareness of the motor deficit [anosognosia for hemiplegia (AHP)], declaring the ability to perform a movement with their left plegic limb, indicative of a sense of agency alteration.

Classical neuropsychological single case studies have reported deficits in the mental representation of the body in deafferented patients (e.g. Refs. ^27–29^), suggesting that central or peripheral proprioception alterations could be the underlying cause for disturbed body and motor awareness, although conflicting findings exist. In the case of body ownership, a recent group study^30^ showed that in patients with stroke, the experience of ownership alterations correlated with the severity of the proprioceptive deficit. Nevertheless, altered ownership with preserved proprioception has also been reported,^31^ questioning the role of proprioception in body awareness. Less is known about the relationship between proprioception deficit and motor unawareness. Prior studies have shown altered agency in patients with proprioceptive impairment. Agency impairment in the context of AHP has been linked to the severity of various neurological symptoms, particularly with the loss of proprioception during the hyperacute phase following a stroke.^32^ This association supports the idea that multiple factors may contribute to AHP,^33,34^ with impaired proprioception potentially playing a key role by hindering the ability to perceive the current state of the paralyzed limb correctly. Differently, Berti *et al.*^35^ observed a double dissociation between AHP and sensory disorders. Some patients with AHP do not exhibit any sensory deficits, while others experience sensory impairments without denying their hemiplegia. To date, no systematic, tailored behavioural and neuroimaging patients’ group study has concurrently investigated the role of proprioception in both body and motor awareness.

**Figure 1:**
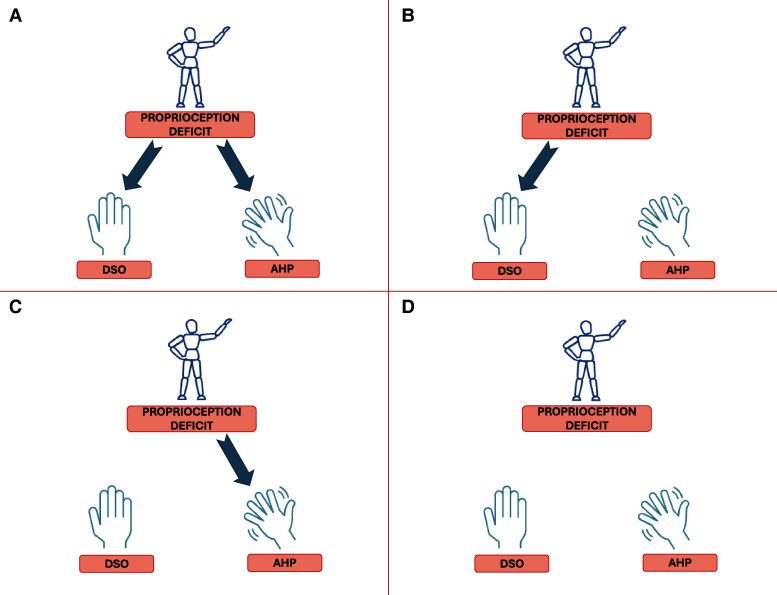
**Study predictions.** Based on existing hypotheses, four study predictions can be made: proprioceptive deficits may predict disturbances in both body ownership (disturbed sensation of ownership—DSO) and agency (anosognosia for hemiplegia—AHP) (**A**); they may selectively predict DSO (**B**); or they may be associated only with AHP (**C**). Alternatively, proprioceptive deficits may not predict disturbances in either DSO or AHP (**D**).

In the current study, we explicitly tested the contribution of proprioception to body and motor awareness, exploring its neural correlates by taking advantage of the specific pathological model of body and motor awareness disturbances and proprioception alterations following right brain damage: DSO, AHP and proprioceptive deficit. Proprioception has been theorized as a non-conceptual and momentary sensory representation, a *condicio sine qua non,* underlying both body and motor awareness. While the congruency between proprioceptive signals, body schema and visual feedback would contribute to the emergence of a coherent sense of ownership, the congruency between proprioceptive signals, motor intention and visual feedback would contribute to a coherent sense of agency.^13^ According to such a conceptualization of proprioception as the sensory root for body and motor awareness, we predict proprioception alterations associated with the presence of both DSO and AHP (Scenario A—Fig. 1). Alternatively, if the Synofzik *et al.*’s postulation is not supported, one might expect that proprioception deficit could be exclusively associated with DSO (Scenario B—Fig. 1), or exclusively associated with AHP (Scenario C—Fig. 1). Lastly, if body and motor awareness are independent from proprioception, proprioceptive deficits would not predict body and motor awareness disorders (Scenario D—see Fig. 1).

From the neural perspective, the neuropsychological approach could offer causal insights into the proprioception contribution to body and motor awareness by exploring the lesional correlates of those proprioceptive alterations, potentially predicting disturbed ownership and agency. Suppose proprioception acts as a root for building body and motor awareness. In that case, we expect that proprioceptive deficits, potentially related to both DSO and AHP, would be linked to lesions to or disconnections between higher-order cerebral areas subserving the multisensory integration at the basis of the sense of the self (i.e. parietal regions^17,36,37^).

## Materials and methods

### Participants

A cohort of 46 individuals was prospectively recruited at the Neurology and Stroke Unit of ASST ‘Grande Ospedale Metropolitano Niguarda’ (Milan, Italy). Eligibility was determined through consecutive admission screening. Participants were required to meet two inclusion criteria: (i) right-handedness and (ii) presence of a right-hemispheric brain lesion confirmed by neuroimaging (CT or MRI). Exclusion criteria comprised: (i) history of psychiatric or neurological disorders, (ii) educational attainment of fewer than 7 years, (iii) ongoing pharmacological treatment with agents known to alter cognition or mood and (iv) language impairments severe enough to interfere with test administration. The study protocol was reviewed and approved by the Ethical Committee ‘Milano Area C’ and adhered to the ethical principles outlined in the Declaration of Helsinki (1964). Written informed consent was obtained from all participants prior to enrolment.

### Neurological and neuropsychological assessment

To obtain an overall measure of cognitive status, patients underwent a brief neuropsychological screening using the Mini-Mental State Examination.^38^ Motor impairment was evaluated through a standardized neurological examination.^39,40^ The severity of hemiplegia was quantified on a four-point scale ranging from 0 to 3, where higher values corresponded to greater motor impairment. A maximum score of 3 denoted the presence of complete hemiplegia.

#### Anosognosia for hemiplegia

Anosognosia for motor deficits was assessed using the standardized five-point scale^39^ previously described in.^41^ Briefly, patients were classified according to their level of awareness, ranging from spontaneous acknowledgment of motor weakness (Score 0) to a complete lack of awareness even after clinical demonstration (Score 4). Only individuals scoring 4 were considered to exhibit complete AHP.

#### Disturbed sensation ownership

Disownership was evaluated for each limb using a standardized four-item procedure previously described in.^41^ The questions addressed ownership, sense of belonging, spatial localization, and ownership attribution of the limb. Responses were transcribed verbatim and scored according to an established system (0 = no disownership, 0.5 = partial disownership, 1 = disownership).^42^ Patients with scores >0 were classified as DSO+. In this cohort, disownership was observed only for the left paralyzed arm/hand.

#### Proprioception

Proprioception was assessed by evaluating verbal reports of static arm postures and imitation with the contralateral limb as in a previous study.^41^ With eyes closed, the examiner placed the affected arm in one of six standardized positions (forward extension with palm up, overhead elevation, lateral extension at shoulder height, resting alongside the body, extension to the right side or military salute). Patients were required to reproduce the posture with the opposite arm and provide a verbal description. Accuracy was scored from 0 to 6, with one point assigned for each correct response.

### Statistical analyses

#### Behavioural

A cumulative link model with proportional odds logistic regression was employed to examine the impact of proprioception scores on DSO and AHP, with hemiplegia as a covariate. The model utilized a logistic distribution with a logit link function to address the distribution of the dependent variable appropriately. To manage infrequent observations commonly seen in studies with rare clinical manifestations (such as DSO and AHP), a bootstrap resampling was also applied to calculate the *P*-values. Data analysis was conducted using JAMOVI and R.

### Lesion mapping

#### Voxel-based lesion-symptom mapping

Voxel-based lesion-symptom mapping (VLSM) was employed to investigate the relationship between lesioned brain tissue and proprioceptive performance. Participants underwent either MRI or CT imaging as part of routine post-stroke clinical evaluation. These scans were subsequently employed for lesion mapping. Lesions were delineated using a semi-automated approach implemented with the Clusterize SPM toolbox,^43–45^ after which the resulting maps were spatially normalized into a standard template through the Clinical toolbox^46^ and resliced for further analyses. Then, the VLSM analyses were conducted on a voxel-wise basis using the MATLAB package NiiStat (https://github.com/neurolabusc/NiiStat). To map the behavioural findings, we used the proprioception predicted values from the behavioural statistical model as dependent variables. To ensure robust statistical inference, we applied a one-tailed threshold of *P* < 0.05 with 5000 permutations, a procedure chosen to maximize statistical power while controlling for family-wise error rates. The directional hypothesis assumed that damaged tissue would be associated with reduced proprioceptive performance. Only voxels affected in at least 10% of participants were included in the analyses. The VLSM analysis was adjusted for lesion volume and the presence of hemiplegia. The anatomical distribution of the grey matter statistical results was assessed using the automated anatomical labelling map (template AAL^47^), which classifies the anatomical distribution of brain images in stereotactic space. Only significant areas with more than ten voxels are discussed.

To assess the regional distribution of the statistical map concerning the white matter damage, we mapped the VLSM results onto tractography reconstructions of white matter pathways obtained from a group of healthy controls.^48^ We quantified the severity of the disconnection by measuring the probability of the tract being disconnected^49^ using Tractotron software as part of the BCBtoolkit^50^; https://storage.googleapis.com/bcblabweb/index.html). Only white matter tracts with a probability of lesion > 0.95 are reported and discussed. This approach was intended to emphasize the most consistently and reliably affected pathways, enhancing anatomical specificity and the interpretability of the findings.

Although our sample size is relatively large for a single-centre study, especially given the low prevalence of these clinical phenomena, it was not sufficient to support reliable subgroup comparisons such as DSO+ versus DSO−. Therefore, we did not perform statistical analyses across subgroups. However, we provided separate lesion overlap maps for the DSO+, DSO− and AHP+, AHP- patients to offer a descriptive comparison (see supplementary materials).

#### Connectome-based lesion symptom mapping

Connectome-based lesion-symptom mapping (CLSM) was employed to investigate the relationship between structural brain connectivity and proprioceptive performance.^51,52^ Normalized and resliced lesion map from the patients were used to estimate the parcel-wise disconnections using the lesion quantification toolkit,^53^ enabling the identification of direct disconnections between grey matter regions for each patient. Lesion quantification toolkit generated structural connectivity matrices by integrating a deterministic HCP842 tractography template^54^ with a functional parcel atlas covering cortical and subcortical grey matter.^55^ For each pair of regions, the number of streamlines disrupted by the lesion was converted to a percentage, producing symmetric 235 × 235 dysconnectivity matrices in which each entry represents the proportion of disconnected streamlines between two regions. These matrices were analysed using a mass-univariate framework to detect associations between structural disconnections and reduced proprioceptive ability. Diagonal and redundant entries were excluded, and only connections affected in at least 10% of the sample were retained. For each ROI-to-ROI connection, a general linear model was computed, with dysconnectivity scores as independent variables and proprioception performance as the dependent variable. Statistical inference was performed using a maximum statistic permutation procedure (*n* = 5000), which generated null distributions for hypothesis testing. Given that most significant VLSM voxels were located in white matter tracts and could affect multiple pathways, we applied a conservative, one-sided significance threshold of *P* < 0.001 to ensure robust detection of meaningful disconnections.

## Results

### Patients sample

The study sample comprised 46 individuals, including 23 females and 23 males, with a mean age of 64.2 years (±15.7) and an average of 10.6 years (±3.98) of formal education. All patients were assessed during the acute phase of stroke, at a mean of 3.6 days (±4.6) from the onset. Global cognitive functioning, as measured by the Mini-Mental State Examination, yielded a mean score of 26.3 (±2.7). The majority of patients in the sample experienced an ischaemic stroke (*n* = 32), while the remaining patients had a haemorrhagic stroke (*n* = 14). Among the 46 individuals included in the study, 23 exhibited left-arm hemiplegia. Within this subgroup, 16 patients presented alterations of ownership and agency (9 patients were diagnosed with DSO, while seven presented with AHP). Performance on the proprioception task revealed a mean score of 4.0 (±2.4). Neurological and neuropsychological evaluations were carried out in a single session to ensure a standardized and time-efficient assessment of interrelated symptoms (e.g. DSO, AHP and proprioception deficit). For DSO, AHP and proprioception assessment, the test scores were assigned by two experimenters (GS and GC), both experts in the evaluation of patients during the acute phase of stroke. Any scores deemed ambiguous were subsequently reviewed in consultation with neurologists (GB and MS).

### Behavioural results

**Figure 2:**
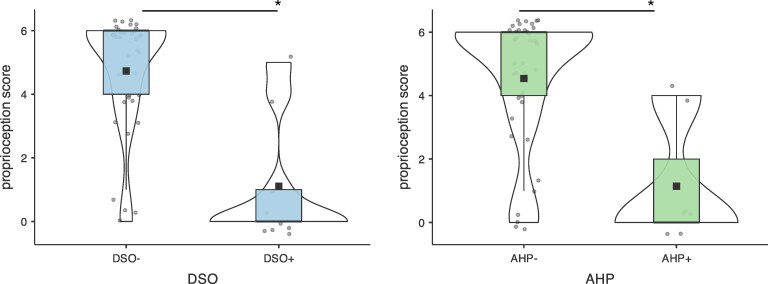
**Behavioural results.** The left graph shows the proprioception scores for patients with and without disturbed sensation of ownership (DSO+ and DSO−, respectively). The right graph shows the proprioception scores for patients with and without anosognosia for hemiplegia (AHP+ and AHP−, respectively). The black squares indicate the median value (50th percentile), while the bounds of the box represent the 25th (lower bound) and 75th percentiles (upper bound), i.e. interquartile range. Jittered points represent the individual data points. Asterisks indicate a significant difference (*P* < 0.05). A cumulative link model with proportional odds logistic regression was employed to examine the impact of DSO and AHP on proprioception scores, with hemiplegia as a covariate in *n* = 46 patients. The model utilized a logistic distribution with a logit link function to address the distribution of the dependent variable appropriately. To manage infrequent observations commonly seen in studies with rare clinical manifestations (such as DSO and AHP), a bootstrap resampling was also applied to calculate the *P*-values.

DSO and AHP were significantly associated with reduced proprioceptive abilities. The overall model was significant (*R*² = 0.2377, *X*²_(3)_ = 33.395, *P* < 0.001). Specifically, proprioceptive abilities were predicted by the presence of DSO (estimate = −2.2319, SE = 0.9500; 95% CI [−4.0938, −0.3699], *z* = −2.3493, *P* = 0.019), with an odds ratio of 0.1073 [95% CI (0.0167, 0.6908)]. Estimated marginal means analysis indicated that individuals without DSO had a higher expected proprioception score [*M* = 3.9784, SE = 0.42057, 95% CI (3.1542, 4.8027)] compared to those with DSO [*M* = 2.2908, SE = 0.61116, 95% CI (1.0930, 3.4887)]. See Fig. 2.

Similarly, the presence of AHP predicted proprioception scores [estimate = −2.1249, SE = 1.0286; 95% CI (−4.1409, −0.1088), *z* = −2.0657, *P* = 0.039], with an odds ratio of 0.1194 [95% CI (0.0159, 0.8969)]. Estimated marginal means analysis indicated that individuals without AHP had a higher expected proprioception score [*M* = 3.9319, SE = 0.36318, 95% CI (3.2201, 4.6437)] than those with AHP [*M* = 2.3374, SE = 0.67007, 95% CI (1.0241, 3.6507)]. See Fig. 2.

To address the unbalanced observations in the DSO and AHP predictors [limited number of patients diagnosed with disorders of ownership and agency (*n* = 16)] and verify the robustness of the results, bootstrap resampling based on distribution’s quintiles with 1000 replicates stratified by DSO and AHP was employed to estimate 95% bootstrap confidence intervals for the main effect of the predictors. Results from bootstrap resampling confirmed that the presence of DSO (*P* = 0.016) and AHP (*P* = 0.026) significantly predicted lower proprioception scores.

### VLSM results

The behavioural findings have demonstrated that patients with DSO and AHP exhibit lower proprioception scores, independent of hemiplegia. To identify brain lesions linked to reduced proprioception (predicting body and motor unawareness), we utilized a VLSM approach. A voxel-wise *t*-test was performed to examine the relationship between lesion status (lesioned or spared) and continuous proprioception scores, using the same dependent variable as in the behavioural analyses. By adjusting for the model estimates of DSO and AHP, this approach allowed us to mirror the behavioural findings in the neuroimaging data. To control for the potential confounding effect of overall damage severity, the presence of hemiplegia and lesion volume were included as covariates. For two patients, the CT scan was not available, and thus the final sample was composed of 44 participants.

**Figure 3:**
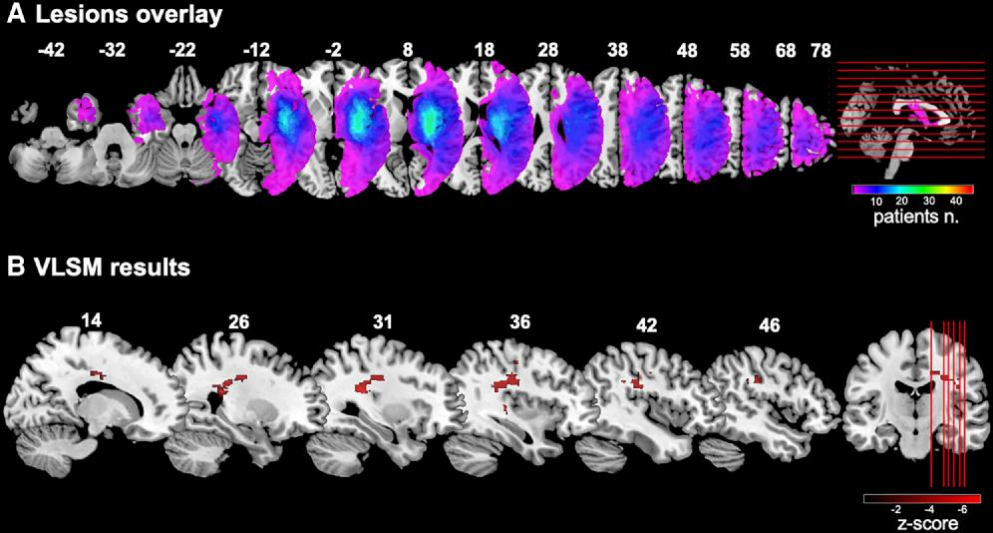
**Lesion overlay and voxel-based lesion symptom-mapping (VLSM) results.** (**A**) The lesion overlay for 44 patients included in the study is shown. The highest lesion overlap (*n* = 24) corresponded to the MCA territory within subcortical regions (e.g. putamen). The colour scale illustrates the corresponding number of lesion overlaps. (**B**) The brain region whose lesion was associated with reduced proprioception ability in all patients is shown. Results are thresholded at *P* < 0.05 and shown on representative sagittal slices of the Montreal Neurological Institute brain. Z-coordinates of each sagittal slice are given. In each slice, the right hemisphere is on the right side. The colour scale illustrates the corresponding Z values. The VLSM analyses were conducted on a voxel-wise basis using the MATLAB package NiiStat (https://github.com/neurolabusc/NiiStat). To map the behavioural findings (*n* = 44 patients), we used the proprioception predicted values of the general linear model as dependent variables. A voxelwise approach was adopted, considering the whole sample. We adopted a one-tailed .05 alpha threshold with 5000 permutations. The permutation-based correction was applied to maximize power while maintaining control of the family-wise error rate (FWER). The one-tailed hypothesis predicted that injured tissue only causes poorer performance, that is, lower proprioceptive ability, a pattern we know from the behavioural model to be associated with DSO+ and AHP+.

The lesion overlay analysis revealed the highest patient’ lesion overlap (*n* = 24) within the middle cerebral artery (MCA) territory, particularly in subcortical regions like the putamen (see Fig. 3A). The VLSM identified 3338 significant voxels, including those in the postcentral gyrus (*n* = 74), supramarginal gyrus (*n* = 38), precentral gyrus (*n* = 29) and Rolandic operculum (*n* = 25). Most of the lesioned voxels were in the white matter, involving the arcuate anterior segment (probability = 1), corpus callosum (probability = 1), frontal commissural tract (probability = 1), superior longitudinal fasciculus I (probability = 0.98), II (probability = 1) and III branch (probability = 1), corticospinal tract (probability = 0.99), fronto-insular tract (probability = 0.98), fronto-striatal tract (probability = 0.96) and Handif U tract (probability = 0.96) (see Fig. 3B).

### CLSM results

**Figure 4:**
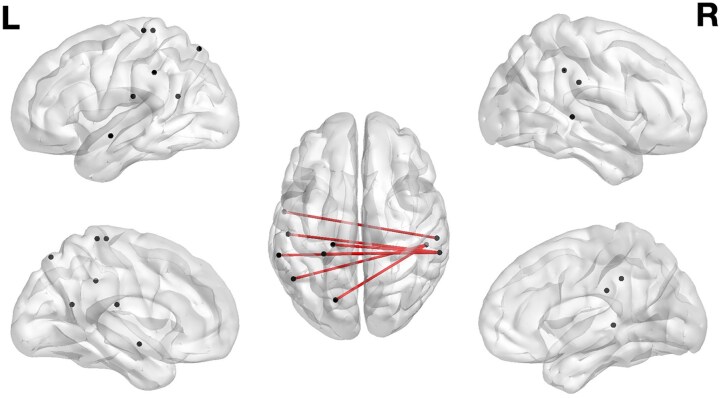
**Connectome-based lesion symptom-mapping results.** Parcel-to-parcel disconnections following the region-to-region disconnectivity analysis. The image presents the disconnections significantly associated with a low proprioception performance following the permutation correction for multiple comparisons (10 000 permutations, corrected *P* < 0.01). Dots indicate regions at the endpoints of significant disconnections. We computed a general linear model for each ROI-to-ROI connection with the dysconnectivity score as the independent variable and the temperature and thermoception indices as dependent variables in two separate analyses (*n* = 44 patients). Then, the maximum statistic permutation (*n* = 5000) was employed on the permuted behavioural data and the original disconnection data with the same analysis strategy to assess the distribution of maximum statistics under the null hypothesis.

We employed CLSM to identify structural disconnections between brain regions associated with lower proprioception scores. CLSM data analyses were performed using the same proprioception index computed for the VLSM analyses. Mapping proprioception scores to ROI-to-ROI analysis identified seven disconnections (see Fig. 4). The analysis revealed that lower proprioception was associated with the disconnection of three hub-like structures involving different parts of the right supramarginal gyrus and the right middle temporal gyrus, disconnected mainly from their contralateral homologue areas (see Table 1).

**Table 1:** CLSM results

Brain region	*x*	*y*	*z*		Brain region	*x*	*y*	*z*
left mid. temporal gyrus	−56	−6	−12	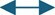	right supramarginal gyrus	60	−26	27
left supramarginal gyrus	−53	−22	18	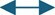	right supramarginal gyrus	62	−37	37
left postecentral gyrus	−26	−38	68					
left postecentral gyrus	−19	−31	68					
left supramarginal gyrus	−60	−39	36					
left sup. parietal gyrus	−17	−73	54	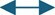	right mid. temporal gyrus	52	−31	2
left mid. temporal gyrus	−48	−57	18					

Parcel-to-parcel disconnections significantly with a low proprioception performance following the region-to-region disconnectivity analysis at *P* < 0.01 (corresponding to Fig. 2). Each row denotes the exact label according to the Schaefer *et al*.^53^ parcellations of each grey matter-to-grey matter disconnection. The anatomical label of the statistical results was assessed using the automated anatomical labelling map (template AAL).

## Discussion

The primary role of proprioception is to stabilize and protect the body. However, its function may extend beyond these fundamental aspects. Emerging theories suggest that proprioceptive signals are at the basis of body and motor awareness. Studies in healthy individuals have found only correlational and partial evidence for this complex relationship by using paradigms (such as the rubber hand illusion), which operationalize proprioception as just one of the body ownership components. Here, we examined the pathological model of patients with right brain damage, with or without proprioceptive alterations, as well as body and motor unawareness.

The classical neuropsychological approach revealed that reduced proprioceptive abilities were associated with DSO and AHP for the contralesional plegic upper limb. Translating this finding into the healthy individual’s framework, and consistent with Synofzik *et al.*’s hypothesis,^13^ this suggests that proprioception is likely to play a crucial role as one of the sensory sources in maintaining a coherent sense of ownership and agency. Although to date, there is no evidence for the relationship between proprioception, body and motor awareness tested in the same brain-damaged patients’ group, these results are in line with the available studies investigating deficits of proprioception and ownership and proprioception and agency separately. For instance, in the framework of proprioception and sense of ownership, evidence indicates that deficits in multisensory integration may contribute to changes in body ownership in stroke patients.^56–58^ However, less is known about the neural correlates of this complex relationship. Only one study^30^ tried to fill this gap by measuring body ownership alterations in stroke patients. Results showed that alteration of ownership in brain-damaged patients results from the combination of proprioceptive and high-level multisensory integration deficits due to frontoparietal damage.

On the other hand, concerning the intricate relationship between proprioception and sense of agency, Vocat and colleagues^32^ demonstrated that proprioceptive loss is strongly correlated with the severity of AHP during the hyperacute phase of stroke. In addition, Balslev *et al.*^59^ tested a chronically deafferented man, comparing him to an age-matched group of six healthy individuals in a task requiring the judgment of the timing of the action. Without proprioception, the patient was less able than the control group to distinguish between their own movements and those of a computer-generated cursor. Similarly, Farrer *et al.*^20^ showed that a haptically deafferented patient, with a loss of proprioception, was impaired compared to the control subjects when asked to match the final position of limbs with an intended movement. Our behavioural neuropsychological findings also make contact with the correlational results in healthy individuals, in which proprioceptive signals could serve as a link in shaping body and motor awareness.^14^

Examining the anatomo-clinical correlations, specifically how lesions and structural disconnections between certain brain regions contribute to impaired proprioception, has offered important insights into the neural substrates involved in processing proprioceptive signals related to body and motor awareness. Specifically, the VLSM has revealed that lesions to grey matter regions (parietal structures such as the primary somatosensory cortex and the supramarginal gyrus) and white matter tracts (SFL, Arcuate, Corticospinal, Fronto-insular and Hand inferior U tract) are associated with lower proprioceptive abilities. The resulting VLSM grey matter regions have been previously associated with body position sense.^60–62^ For instance, an fMRI study^63^ examined how the brain processes information about arm position from both sight (visual) and body sense (proprioception), finding that when both visual and proprioceptive signals match, specific brain areas, including the parietal cortex, are activated. Furthermore, previous evidence investigating the role of white matter disconnections in the emergence of proprioceptive deficits has highlighted that lesions to the superior longitudinal fasciculus (SLF II and III), arcuate fasciculus (all segments) and fronto-insular tracts were associated with more severe proprioceptive deficits.^64^ Interestingly, regions highlighted by the VLSM results have also been previously identified as the specific substrate for body and motor awareness. Specifically, the importance of the parietal lobe in motor awareness has been supported by several neuropsychological studies and functional neuroimaging experiments, which highlighted its significant role in how individuals are aware of their own motor functions.^65–67^ Neurological conditions such as AHP have been linked to abnormalities in the frontoparietal networks.^68–71^ In a seminal lesion mapping study, Berti *et al.*^68^ found that AHP was linked to lesions in motor and somatosensory areas. Later studies have also emphasized the importance of insular regions and white matter tracts.^33,72^ Pacella *et al.*^73^ showed that white matter disconnections may also play a role in AHP, involving disruptions in the cingulum, SFL III and frontal aslant. Also, disturbances in the sense of ownership have been linked to abnormalities in the parietal lobe, within wider cortical networks and white matter tracts.^26,74^ Specifically, DSO has been shown to be associated with lesions to the supramarginal gyrus and disconnections of a fronto-insular-parietal network involving the frontal-insular and frontal inferior longitudinal tracts.^75^ Our findings also align with previous fMRI studies in healthy individuals, which identified the resulting cerebral regions as crucial for proprioception, body, and motor awareness. For instance, an event-related fMRI study^62^ confirmed the laterality of the right hemisphere in proprioceptive abilities, pointing out the role of the right supramarginal gyrus as a critical region whose activity was reduced in the presence of proprioceptive deficits. The authors also found the dorsal premotor cortex to be part of proprioception-related brain activation. In a more recent fMRI meta-analysis, Kenzie and colleagues^76^ showed that proprioception is related to the activation of the left precentral, postcentral, and anterior cingulate gyrus. Notably, distinct activation patterns emerged depending on the proprioception measure. In contrast to the above-mentioned findings in healthy and pathological populations, particularly those implicating the premotor area in disturbances of body and motor awareness, our analyses did not reveal involvement of this region. This absence, however, may be expected given that our investigation focused specifically on proprioceptive deficits rather than on DSO or AHP per se.

In our study, white matter damage linked to proprioceptive impairments indicated that regions supporting the role of proprioception in the sense of ownership and agency might extend beyond the sites of direct tissue injury. Specifically, CLSM analysis showed that reduced proprioceptive function (observed in patients with DSO and AHP) was mainly related to interhemispheric disconnection in dorsal areas involved in multisensory integration, which is essential for bodily self-awareness.^17,36^ These regions include the left and right parietal and the temporal lobes. The role of the left and right hemisphere regions in proprioception has also been highlighted by an fMRI metanalysis of proprioception in healthy individuals. For instance, the metanalytic results on the brain activations following passive movements have shown clusters involving the left and right parietal lobes (left pre- and post-central gyri and right inferior parietal lobule). Thus, unilateral proprioception is likely supported by inter-hemispheric communication predominantly involving the corpus callosum.^15^ An alternative explanation for our bilateral disconnection findings could involve the task demand. In our study, patients were asked to reproduce the position of the affected arm by using the healthy limb. This might have induced a crosstalk between the two hemispheres (bimanual task), in which patients with absent proprioception may fail.

By bringing together our findings that have shed light on the hodological and topological components^77^ associated with deficits in body and motor awareness due to proprioceptive damage, we can speculate on the neurocognitive mechanisms underlying bodily self-awareness. A robust finding is represented by lesions affecting the sensorimotor areas, accompanied by disconnections caused by the underlying white matter (e.g. superior longitudinal fasciculus). Such a lesional pattern has already been described in the case of DSO and AHP.^41,68,75,78^ However, an additional critical factor emerges here, involving not only unilateral damage to the fasciculus connecting parietal and premotor areas but also bilateral disconnection of the parietal and postcentral cortices. We can therefore hypothesize that the clinical association often observed between DSO and AHP stems from a multi-level lesion pattern that results in the degradation of proprioceptive signals. These signals fail to be adequately integrated within the sensorimotor circuits of the lesioned hemisphere. Importantly, this integration appears to require coordinated activity between the parieto-temporal areas of both hemispheres, which is disrupted in the pathological condition. The bilateral disconnection suggests that interhemispheric communication, possibly mediated by commissural fibres such as the corpus callosum, is crucial for maintaining coherent body awareness (see also Refs ^41,78^). Besides, while this study lends support to the hypothesis of Synofzik *et al.*,^13^ further targeted experiments are warranted. A suitable approach could involve a paradigm that concurrently examines the sense of ownership and the sense of agency in patients with and without proprioception, enabling a more refined and hypothesis-driven investigation.

In conclusion, this study provides novel insights into the integral role of proprioception in body and motor awareness, an area where behavioural investigations in healthy populations have yet to yield definitive evidence. Through the integration of classical neuropsychological behavioural assessments and lesion-symptom mapping, we offer causal evidence that contributes significantly to theoretical frameworks of bodily self-awareness. The proprioceptive system exhibits considerable plasticity, continuously adapting to recurrent postural changes through ongoing sensorimotor updates. The effective integration of these updates is essential for the maintenance of coherent body and motor awareness. Disruption of this integrative process due to cerebral lesions may result in deficits of coherent self-awareness, thereby facilitating the manifestation of disorders such as DSO and AHP. These findings have important implications for the development of targeted rehabilitative interventions, including proprioceptive training protocols, applicable across diverse patient populations exhibiting disturbances in the sense of ownership and agency. Notably, prior experimental evidence in healthy individuals demonstrated that enhancement of proprioceptive inputs can mitigate alterations induced by paradigms such as the rubber hand illusion.^79^ Lastly, the neural systems we identified here could potentially serve as anatomical stimulation targets for non-invasive instrumental rehabilitation.

## Supplementary Material

fcaf379_Supplementary_Data

## Data Availability

The data generated during this study are available under restricted access due to the inclusion of sensitive information (e.g. age, gender, education level and admitting hospital) that could potentially compromise participant privacy and consent. Access to the data can be requested from the corresponding author (gerardo.salvato@unipv.it), upon approval.
