# Supplementary material for: Proprioception as a sensory root for body and motor awareness
Source: Brain Commun. 2025 Oct 1;7(5):fcaf379. doi: 10.1093/braincomms/fcaf379 (PMC12541373; doi:10.1093/braincomms/fcaf379)
Supplement: fcaf379_Supplementary_Data [file fcaf379_supplementary_data.pdf]

Supplementary materials for:

Proprioception as a sensory root for body and motor awareness

Gerardo Salvato[1,2,3*], Giulia Casile[1], Silvia Amaryllis Claudia Squarza[4], Mariangela Piano[4], Maria Sessa[5], Gabriella Bottini[1,2,3]

[1] Department of Brain and Behavioral Sciences, University of Pavia, Pavia, 27100, Italy.
[2] Cognitive Neuropsychology Centre, ASST "Grande Ospedale Metropolitano Niguarda", Milano, 20162, Italy.
[3] NeuroMi, Milan Center for Neuroscience, Milano, 20126, Italy.
[4] Neuroradiology Unit, ASST "Grande Ospedale Metropolitano Niguarda", Milano, 20162, Italy.
[5] Neurology and Stroke Unit, ASST "Grande Ospedale Metropolitano Niguarda", Milano, 20162, Italy.

Supplementary figure

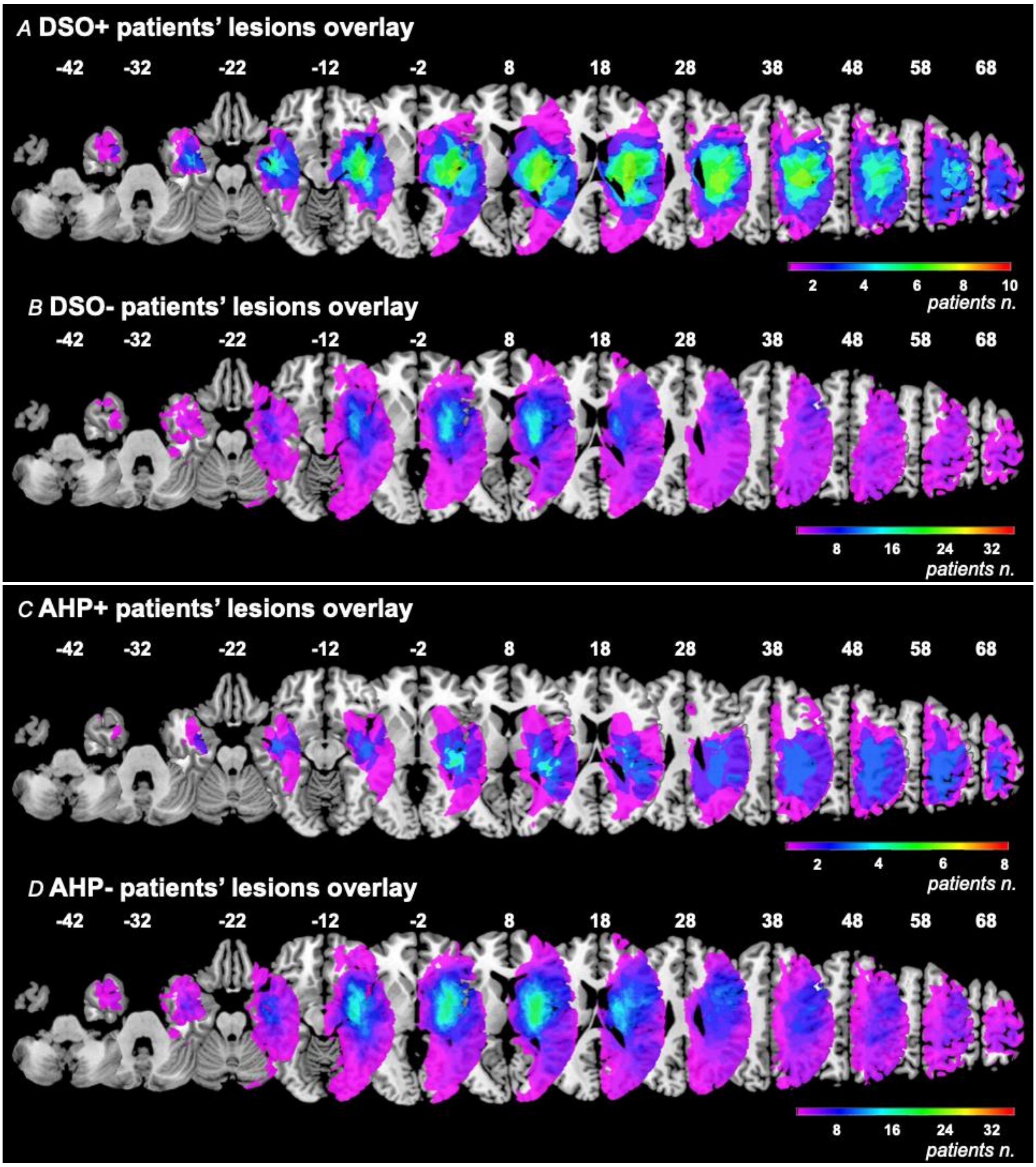

Supplementary Figure 1. **Lesion overlay.** Panels A and B display lesion overlap maps for patients with (DSO+) and without (DSO–) Disturbed Sensation of Ownership, respectively. Panels C and D show lesion overlays for patients with (AHP+) and without (AHP–) Anosognosia for Hemiplegia. The color scale represents the number of overlapping lesions across patients. In each axial slice, the right hemisphere is shown on the right. Slice levels are indicated by white numbers.
